# A Rare and Unusual Case of Trisomy 10p with Terminal 14q Deletion: A Multidisciplinary Approach

**DOI:** 10.7759/cureus.15459

**Published:** 2021-06-05

**Authors:** Chanan Goyal, Vivek Goyal, Waqar M Naqvi

**Affiliations:** 1 Community Physiotherapy, Datta Meghe Institute of Medical Sciences, Wardha, IND; 2 Paediatric Physiotherapy, Government Physiotherapy College, Raipur, IND; 3 Anaesthesiology, Shri Balaji Institute of Medical Science, Raipur, IND

**Keywords:** trisomy 10p, terminal 14q deletion, goyal-naqvi syndrome, rare genetic disorder, neurodevelopmental treatment, sensory integration

## Abstract

Trisomy 10p is a rare entity to be diagnosed and so is terminal 14q deletion. The total number of trisomy 10p cases reported to date is estimated to be in double digits. The number of terminal 14q deletion cases that have been reported in the literature is even lesser than that of trisomy 10p. Simultaneous occurrence of these genetic aberrations is, therefore, extremely rare. Herein, we document a case of a 14-month-old female diagnosed with trisomy 10p and terminal 14q deletion, who presented with an inability to sit without support and had difficulty in holding her neck. She had no means of independent indoor mobility, which was further limiting her development by exploration. Clinical features included hypotonia, developmental delay, extraneous movements of the head and tongue, intellectual impairment, and facial dysmorphism. She could maintain tripod sitting for less than a minute. Physiotherapy intervention was based on principles of neurodevelopmental treatment and sensory integration. After nine months of physiotherapy intervention, her total gross motor function measure (GMFM) score improved from 11% to 40%. The functional gains were maintained with a home exercise program, after almost one year of discontinuation of institution-based physiotherapy. To the best of our knowledge, this is the first report on the management of a child with the diagnosis of trisomy 10p along with terminal 14q deletion. Further research on the role of early intervention to maximize functional potential in rare genetic conditions is warranted.

## Introduction

Trisomy 10p is a rare entity to be diagnosed and so is terminal 14q deletion. In 1974, trisomy 10p was documented for the first time as a novel syndrome in a pair of siblings [1]. According to a study published in December 2011, 58 cases of trisomy 10p had been documented by then [2]. The total number of trisomy 10p cases reported to date is estimated to be in double digits, but trisomy 10 is relatively easier to find. Around 20 patients of terminal 14q deletion have been reported in the literature as per a study published in 2009 [3]. The first description of terminal 14q deletion syndrome dates back to 1997, although interstitial deletions have been documented even earlier [4]. Unlike trisomy 10p, terminal 14q deletion is not associated with multiple congenital anomalies [3]. Nevertheless, intellectual impairment, peculiar facies, and global developmental delay with a stable general condition are common to both conditions. Simultaneous occurrence of genetic aberrations in the short arm of chromosome 10 and the long arm of chromosome 14 is extremely rare. One previous study has documented trisomy of the short arm of chromosome 10 along with translocation that largely comprised of the long arm of chromosome 14 and the short arm of chromosome 10 [5]. To the best of our knowledge, as we present the index case diagnosed as trisomy 10p along with terminal 14q deletion, the rehabilitation of a patient with this unique combination of genetic anomalies is thus reported for the first time herein.

## Case presentation

Patient information

A 14-month-old female presented to the department of physiotherapy with a major concern of her parents being her frequent throwing back of head and inability to sit without support. As per her parents, she was the firstborn of a non-consanguineous marriage. There was no history of previous pregnancies or miscarriages. The father was 32 years old and the mother was 30 years old at the time of her birth. An ultrasound scan done in the third trimester revealed swelling in the left kidney. The pregnancy was otherwise uneventful and she was delivered at full term through caesarean section. After birth, she cried immediately and weighed 2.6 kilograms. Thereafter, they noticed diminished spontaneous movements during early infancy. There was no family history of developmental delay in up to the second-degree relatives on the maternal or paternal side. The child was presented to a paediatrician at seven months of age who suggested vitamin D supplementation and detailed workup including genetic tests for the diagnosis of the condition. At the age of 14 months, she was presented to a paediatric physiotherapist.

Clinical findings

**Figure 1:**
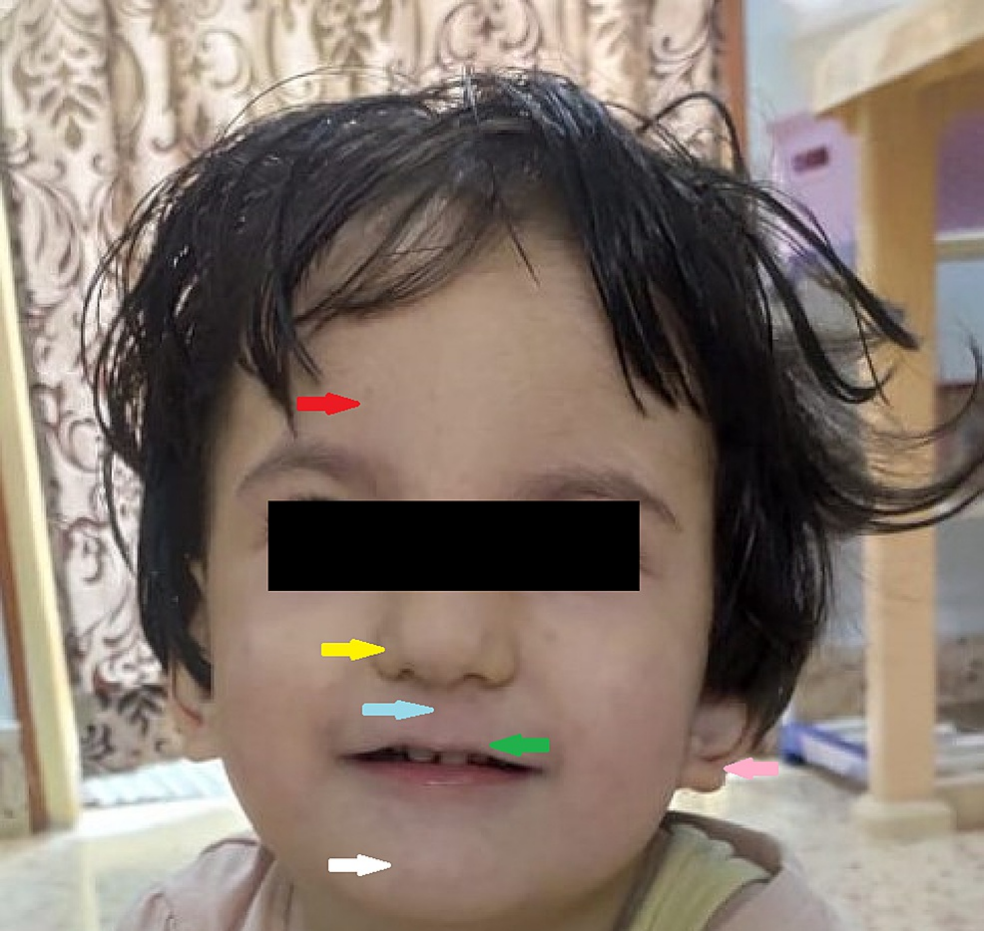
Facial dysmorphism The red arrow points to a prominent forehead, the yellow arrow points to a bulbous nose, the blue arrow points to a long philtrum, the green arrow points to a thin upper lip vermilion and narrow mouth, the pink arrow points to low-set ears, and the white arrow points to retrognathia.

On observation, she had dysmorphic facial features including long face, frontal bossing, brachycephaly, hypertelorism, downward slant of palpebral fissures, bulbous nose with wide nasal bridge, long philtrum, thin upper lip vermilion, mild retrognathia, narrow mouth, and low-set ears (Figure 1).

**Figure 2:**
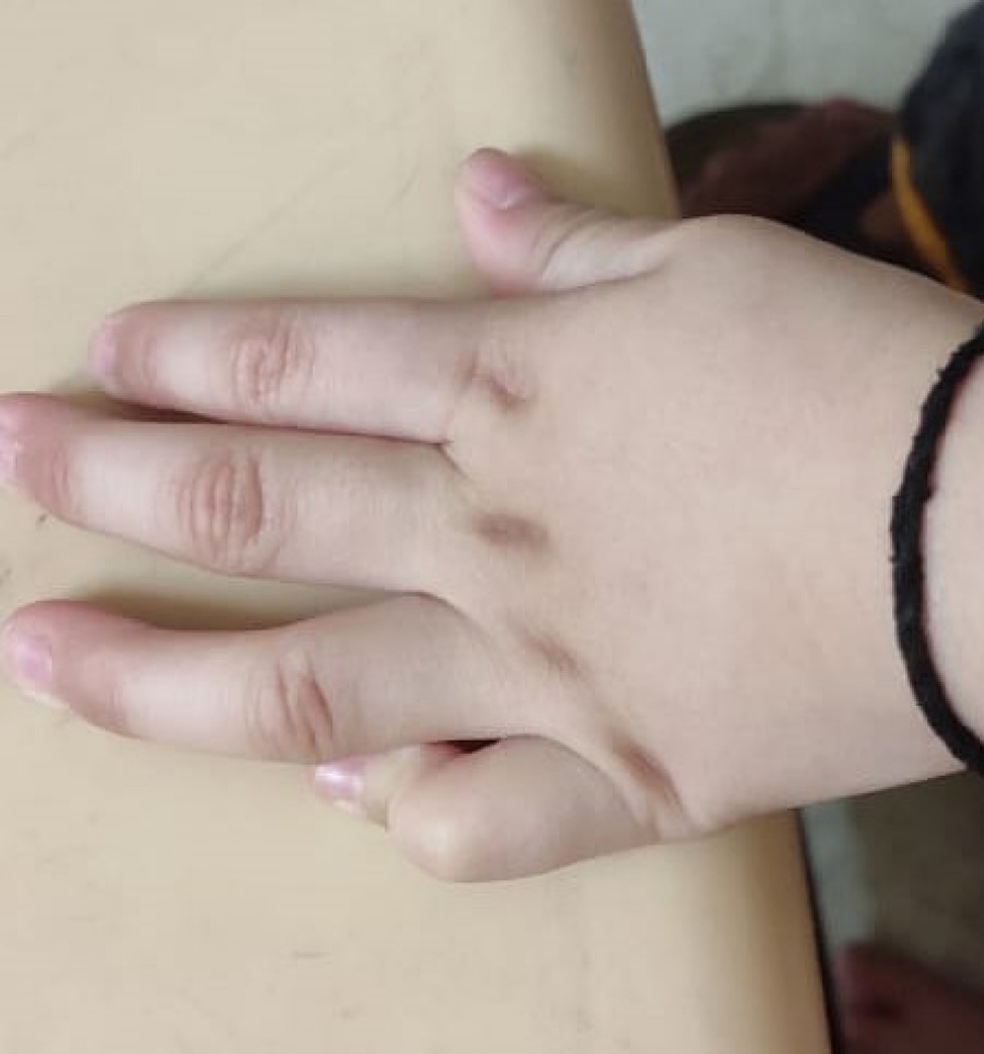
Camptodactyly (left little finger)

Camptodactyly in the left little finger was also observed (Figure 2).

**Figure 3:**
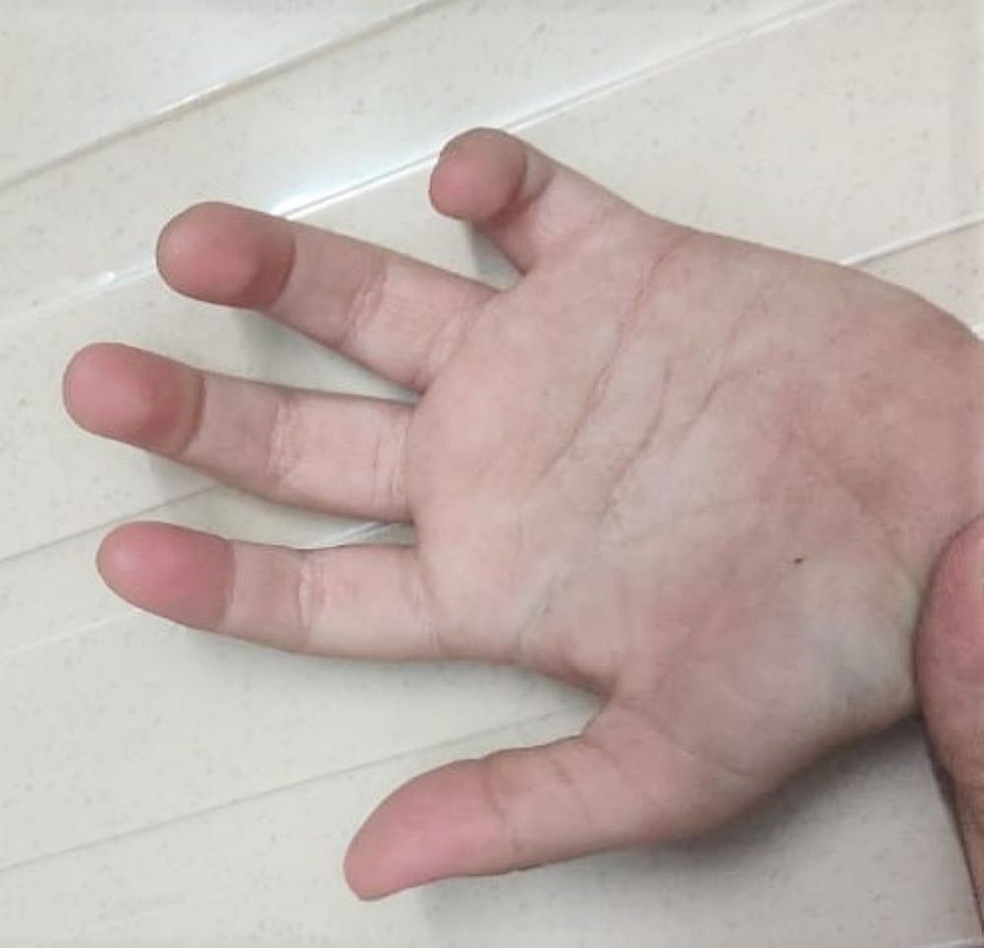
Atypical palmar creases

She also displayed atypical palmar creases (Figure 3).

**Figure 4:**
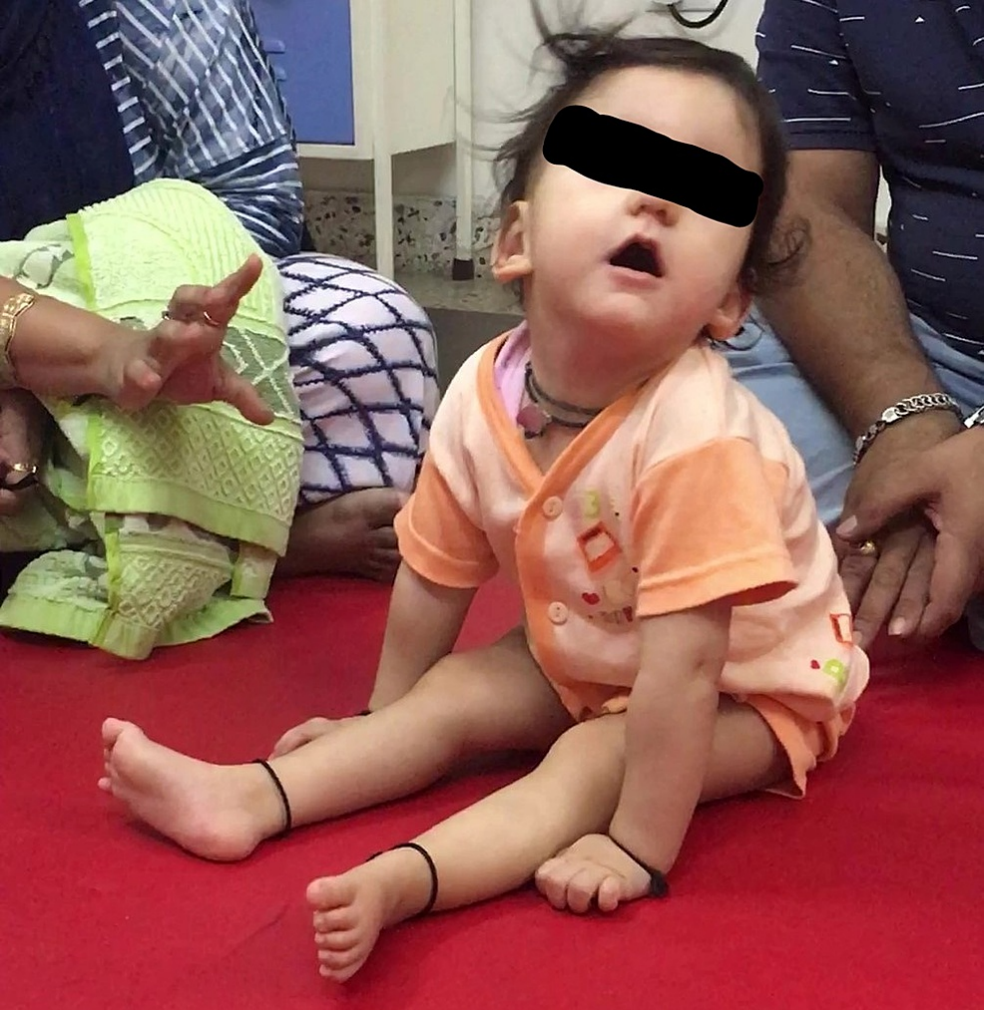
Tripod sitting at the age of 14 months (before intervention)

On examination, she had generalized hypotonia and weakness of the trunk muscles. Bilateral knee hyperextension was noted while standing with maximum assistance. When made to sit, she could maintain tripod sitting, with widely abducted hips forming a large base of support, for less than a minute and then fell backwards leading by the head (Figure 4).

She displayed frequent extraneous movements of the head and tongue while sitting with hand support (Video 1).

**Video 1:** Extraneous movements of the head while sitting with hand support The face has been blurred to avoid patient identification.

She was able to perform rollover from prone to supine but not from supine to prone. Her gross motor function measure (GMFM) score was 41% in Dimension A and 13% in Dimension B. Her total GMFM score was 11% [6,7]. She did not attempt reaching and grasping while supported sitting. However, in the supine lying position, she attempted to hold objects with a gross grasp when offered near the hand. Also, her expressive communication was limited to single syllables and her comprehension was grossly limited too.

Diagnostic assessment

Magnetic resonance imaging (MRI) of her brain showed no demonstrable abnormality. Ultrasound of abdomen was suggestive of mild left hydronephrosis with baggy pelvis. Her chromosomal microarray analysis (CMA) indicated trisomy 10p due to duplication of 28.7 Mb at cytoband 10p15.3p12.1 and terminal 14q deletion (heterozygous) of 2.2 Mb at cytoband 14q32.33, encompassing approximately 25 genes, including MTA1 and AKT1 genes. These genes play a crucial role in neurological development [8,9]. On chromosomal analysis of her mother, a karyotype with a balanced translocation between chromosome 10 and chromosome 14 was found.

Therapeutic intervention

The primary goal was to provide a safe mode of independent indoor mobility so that the movement helps the child in play and exploration of her environment. Physiotherapy intervention was based on the neurodevelopmental treatment approach that derives from the principles of typical development and motor learning. Rolling facilitation was practised on the Swiss ball and on a mat. Transitions like supine to sitting through side-lying, sit to stand, and half kneel to stand were facilitated by an appropriate hand placement of the therapist. Static and dynamic sitting balance was challenged by performing activities like multidirectional reaching for objects of interest while using equipment like Swiss ball, equilibrium board, and astronaut board. Standing with support to play a ball game and reaching out while crossing midline were included as weight-bearing exercises. Cruising along furniture was facilitated. Walking facilitation was practised using a walker (rollator) and a partial body-weight-support manual harness. Pushing a Swiss ball and pulling a theraband with assistance was practised while 90-90 sitting with support. The sensory integration approach was used to address extraneous movements of the head and the tongue. Oromotor vibration through an oral therapy tool and gentle compressions to temporomandibular joints were imparted. Deep pressure and joint compressions were imparted for proprioceptive input to increase the awareness of the body’s position. Vestibular stimulation was provided through a swing system with safety precautions with an intent to satiate the craving for movement and to improve balance. Trunk stabilizing pressure input orthosis (SPIO) was used during active participation in tasks. Hand functional training included holding onto a cup for drinking, eating finger food, and scribbling.

Follow-up and outcomes

After nine months of physiotherapy intervention for five times a week, her GMFM score was 80% in Dimension A, 72% in Dimension B, 19% in Dimension C, 13% in Dimension D, and 15% in Dimension E. Her total GMFM score was 40%. She was able to sit without support and move independently indoors by scooting (Video 2). When provided with manual support, she was able to walk with both hands held. Extraneous movements of the head and tongue were seldom observed. Mild knee hyperextension on weight-bearing was present bilaterally.

**Video 2:** Scooting as means of independent indoor mobility and exploration of objects at the age of 23 months (after intervention)

Owing to the COVID-19 pandemic situation in India, from March 2020 onwards, institution-based physiotherapy sessions were discontinued and the parents were educated on a home exercise program. In February 2021, the parents presented the child to the paediatric physiotherapist on a virtual platform. In the online consultation, it was observed that the child used scooting as a mode of indoor movement comfortably. She could walk with hands held but with bilaterally pronated feet (Video 3).

**Video 3:** Supported walking with bilaterally pronated feet at the age of 36 months

Besides, she attempted cruising along the furniture. Also, she was actively participating in self-feeding the finger food using a palmar grasp. Her speech was limited to bisyllables and she tried to communicate by producing sounds. However, as per the parents, that the child had some understanding of gestures but the comprehension of spoken language was uncertain. Telerehabilitation sessions and footwear with medial arch support were recommended in addition to the home exercise program. The timeline of events is summarized in Table 1. 

**Table 1:** Timeline of events GMFM: gross motor function measure

Date	Consultation	Event/Test	Diagnosis/ Findings	Suggestions/ Treatment
15 February 2018	Obstetrician and Paediatrician	Birth	-	-
17 August 2018	Paediatrician	Clinical assessment	Global developmental delay and peculiar facies	MRI of brain, metabolic and genetic work-up
21 September 2018	Radiologist	Magnetic Resonance Imaging of Brain	No demonstrable abnormality	-
22 November 2018	Biochemical geneticist	Tandem Mass Spectroscopy	Normal level of acylcarnitines and amino acids	-
31 December 2018	Cytogenecist	Chromosomal Microarray Analysis	Trisomy 10p and Terminal 14q32.33 Deletion	Genetic Counselling
5 February 2019	Radiologist	Ultrasound of Abdomen	Left hydronephrosis with baggy pelvis	-
5 February 2019	Clinical Pathologist	Complete Blood Picture	Hypochromic anemia	Iron and folic acid supplementation
6 April 2019	Paediatric Physiotherapist	GMFM score	Dimension A 41%, Dimension B 13% GMFM total score- 10.8%	Neurodevelopmental Treatment and Sensory Integration
2 February 2020	Paediatric Physiotherapist	GMFM score	Dimension A 80%, dimension B 72%, dimension C 19%, dimension D 13%, dimension E 15% GMFM total score- 40%	Home exercise plan and regular follow up
6 February 2021	Paediatric Physiotherapist	Online Reassessment	Motor function maintained	Telerehabilitation and medial arch support

## Discussion

Clinical manifestations presented in the child were similar to those found in the previous studies. The pioneering study on trisomy 10p syndrome and others following it included severe intellectual impairment, motor developmental delay, dysmorphic facies (including long face, prominent forehead, hypertelorism, and downturned corners of the mouth), low-set ears, hypotonic muscles, joint laxity, atypical palmar creases, camptodactyly, and renal anomaly as the peculiar features, which were also noticed in the case that we document [1,2,10,11]. The commonly found clinical features as documented in the previous studies on terminal 14q deletion syndrome comprise abnormalities of skull shape, frontal bossing, epicanthal folds, wide nasal bridge, long and broad philtrum, micrognathia, hypotonia, global developmental delay, and mental retardation, which were also observed in the presented case [3,4,12-16]. GMFM-88 was used for the evaluation of motor skills because its reliability and validity have been established for conditions other than cerebral palsy [6,7]. Physiotherapy management of children with genetic syndromes with tonal abnormalities based on the principles of neurodevelopmental treatment and sensory integration has also been found to be helpful in the literature in improving sensory-motor function [17-19]. Vestibular input helped in improving modulation and in reducing extraneous movements as was observed in former studies on varied causes of developmental delays [18,20]. Home exercise program with the active involvement of child must have helped in retaining the achievement in motor skills. Trunk SPIO has been found to beneficial in a previous study on a child with another rare genetic syndrome characterized by hypotonia [19].

As we document an unusual condition with an emphasis on its management, which has not been published yet, concomitant occurrence of trisomy 10p and terminal 14q deletion can be referred to as Goyal-Naqvi syndrome for the documentation of cases that would be reported hereafter. Nonetheless, whether the same change in motor development could be obtained regardless of physiotherapy is uncertain, as the natural history of the condition is less known and is a definite avenue for future research.

## Conclusions

The child demonstrated remarkable improvement in gross motor function. During the course of rehabilitation, she came a long way from not being able to hold her neck adequately at the age of 14 months to be able to stand and walk with minimal assistance at the age of 23 months. The functional gains were maintained after almost one year of discontinuation of institution-based physiotherapy. Although a home exercise program was followed by her parents at home. This emphasizes the importance of education and empowerment of parents in terms of the management of their child.

Genetic tests to confirm the diagnosis in children with suspected chromosomal anomalies are often not feasible due to the financial constraints of the family, especially in underdeveloped and developing nations. Children with developmental delay should be referred to early intervention even if the definitive diagnosis is pending. The delineation of the phenotype spectrum by documenting characteristic manifestations in multiple patients would facilitate diagnosis. Irrespective of definitive diagnosis, in case of global developmental delay, referral to specialists in the rehabilitation team helps in the holistic development of the child to tap the maximum functional potential.

## References

[1] Schleiermacher E, Schliebitz U, Steffens C (1974). Brother and sister with trisomy 10p: a new syndrome. Humangenetik.

[2] Szabó GP, Knegt AC, Ujfalusi A, Balogh E, Szabó T, Oláh É (2012). Subtelomeric 6.7 Mb trisomy 10p and 5.6 Mb monosomy 21q detected by FISH and array-CGH in three related patients. Am J Med Genet A.

[3] Schlade-Bartusiak K, Ardinger H, Cox DW (2009). A child with terminal 14q deletion syndrome: consideration of genotype-phenotype correlations. Am J Med Genet A.

[4] Ortigas AP, Stein CK, Thomson LL, Hoo JJ (1997). Delineation of 14q32.3 deletion syndrome. J Med Genet.

[5] Hustinx TW, Ter Haar BG, Scheres JM, Rutten FJ (1974). Trisomy for the short arm of chromosome No. 10. Clin Genet.

[6] Michaelis U (2015). Gross Motor Function Measure (GMFM-66 & GMFM 88) User's Manual 2nd Edition Clinics in Developmental Medicine edited by Russell, Dianne J, Rosenbaum, Peter L, Wright, Marilyn, Avery, Lisa M London, UK: Mac Keith Press, 2013 £70.00 (Spiral Binding), pp 290 ISBN: 978-1-908316-88-2. Dev Med Child Neurol.

[7] Harvey AR (2017). The Gross Motor Function Measure (GMFM). J Physiother.

[8] Kumar AS, Jagadeeshan S, Subramanian A (2016). Molecular mechanism of regulation of MTA1 expression by granulocyte colony-stimulating factor. J Biol Chem.

[9] Shumay E, Wiers CE, Shokri-Kojori E (2017). New repeat polymorphism in the <i>AKT1</i> gene predicts striatal dopamine D2/D3 receptor availability and stimulant-induced dopamine release in the healthy human brain. J Neurosci.

[10] Courtens W, Wuyts W, Scheers S (2006). A de novo subterminal trisomy 10p and monosomy 18q in a girl with MCA/MR: case report and review. Eur J Med Genet.

[11] Dabir TA, Morrison PJ (2006). Trisomy 10p with clinical features of facio-auriculo-vertebral spectrum: a case report. Clin Dysmorphol.

[12] van Karnebeek CD, Quik S, Sluijter S, Hulsbeek MM, Hoovers JM, Hennekam RC (2002). Further delineation of the chromosome 14q terminal deletion syndrome. Am J Med Genet.

[13] Maurin ML, Brisset S, Le Lorc'h M (2006). Terminal 14q32.33 deletion: genotype-phenotype correlation. Am J Med Genet A.

[14] Bağci G, Cetin GO, Semerci N, Toruner GA, Cinbiş M (2012). Terminal 14q deletion with unbalanced t(Y;14)(q12;q32) translocation. Clin Dysmorphol.

[15] Schneider A, Benzacken B, Guichet A (2008). Molecular cytogenetic characterization of terminal 14q32 deletions in two children with an abnormal phenotype and corpus callosum hypoplasia. Eur J Hum Genet.

[16] Chen CP, Ko TM, Chen YY (2017). Prenatal diagnosis and molecular cytogenetic characterization of low-level mosaicism for tetrasomy 18p at amniocentesis in a pregnancy with a favorable outcome. Taiwan J Obstet Gynecol.

[17] Goyal C, Naqvi WM, Sahu A (2020). An atypical case of febrile infection-related epilepsy syndrome following acute encephalitis: impact of physiotherapy in regaining locomotor abilities in a patient with neuroregression. Pan Afr Med J.

[18] Goyal C, Naqvi W, Sahu A (2020). Xia-Gibbs syndrome: a rare case report of a male child and insight into physiotherapy management. Cureus.

[19] Goyal C, Naqvi WM, Sahu A, Aujla AS (2020). Xia-Gibbs syndrome: a review of literature. Cureus.

[20] Goyal CV, Naqvi WM (2020). Lordoscoliosis and hyperlordosis in quadriplegic cerebral palsy. Pan Afr Med J.

